# Critical review on data processing algorithms in non-target screening: challenges and opportunities to improve result comparability

**DOI:** 10.1007/s00216-023-04776-7

**Published:** 2023-06-29

**Authors:** Gerrit Renner, Max Reuschenbach

**Affiliations:** 1 Instrumental Analytical Chemistry, University of Duisburg-Essen, Universitätsstr. 5, Essen, D-45141 NRW Germany; 2 Centre for Water and Environmental Research (ZWU), University of Duisburg-Essen, Universitätsstr. 2, Essen, D-45141 NRW Germany

**Keywords:** Non-target screening, Data processing, Algorithms, Challenges

## Abstract

Non-target screening (NTS) is a powerful environmental and analytical chemistry approach for detecting and identifying unknown compounds in complex samples. High-resolution mass spectrometry has enhanced NTS capabilities but created challenges in data analysis, including data preprocessing, peak detection, and feature extraction. This review provides an in-depth understanding of NTS data processing methods, focusing on centroiding, extracted ion chromatogram (XIC) building, chromatographic peak characterization, alignment, componentization, and prioritization of features. We discuss the strengths and weaknesses of various algorithms, the influence of user input parameters on the results, and the need for automated parameter optimization. We address uncertainty and data quality issues, emphasizing the importance of incorporating confidence intervals and raw data quality assessment in data processing workflows. Furthermore, we highlight the need for cross-study comparability and propose potential solutions, such as utilizing standardized statistics and open-access data exchange platforms. In conclusion, we offer future perspectives and recommendations for developers and users of NTS data processing algorithms and workflows. By addressing these challenges and capitalizing on the opportunities presented, the NTS community can advance the field, improve the reliability of results, and enhance data comparability across different studies.

## Introduction

Organic micropollutants of partly unknown origin, fate, and chemical behavior enter our aquatic environment by various routes [1]. To address these blind spots and mitigate human and ecotoxicological risks, Brunner et al. and numerous other research groups have highlighted the increasing importance and need for comprehensive analytical monitoring strategies. These approaches aim to account for virtually all known and unknown organic compounds in environmental samples [2]. The non-target screening (NTS) is one of the most promising methods for this analytical issue, and many of its aspects, e.g., sampling, sample preparation, or measurement methods, are continuously developed and improved.

NTS was initially established as a qualifying method that does not require reference standards by design. Substances not yet covered by target analysis or suspect screening can be detected via NTS, and after successful identification, the suspect list can be expanded. NTS is commonly used for samples with unknown and variable substance compositions like waste waters or rivers. This can be obtained, among others, from Purschke et al., who investigated industrial wastewater [3], and from Tisler et al., who analyzed urban wastewaters [4]. However, the compromise that has to be made with NTS is that, in many cases, reliable identification of unknown substances cannot be made due to the very high number of substance signals often combined with poor signal quality. In addition to qualification methods, the first quantification approaches are currently being evaluated, which is well demonstrated in the work of Malm et al. and Aalizadeh et al. [5, 6].

NTS detects abstract features of substances within the measurement data not covered by conventional target analysis or suspect screenings. Therefore, NTS is an upcoming technology for modern risk mitigation and to fulfill the precautionary principle of the European Union [7]. Naturally, NTS features depend on the analytical instrument domains. One widely used technique is high-performance liquid chromatography with high-resolution mass spectrometry (HPLC-HRMS). This method generates feature triplets consisting of retention time (rt), mass-charge ratio (m/z), and intensity (I).

A typical LC-HRMS dataset comprises a series of mass spectra collected over time, reflecting the separation of analytes by the chromatographic system. These mass spectra are recorded independently, meaning that individual m/z values may differ. Consequently, the data structure is not a large matrix with predefined time and m/z domain channels but rather a collection of high-resolution mass spectra that require processing to obtain XICs and identify features. In this data structure, the extracted ion chromatograms represent the intensity of a specific m/z value across the chromatographic time domain. XICs provide information on the elution profile of analytes and can be used for peak detection and integration. Charge state dispersion is another important aspect of LC-HRMS data, as it refers to the distribution of various charge states that an ion can adopt in the mass spectrometer. The presence of multiple charge states can complicate data interpretation and provide valuable information for identifying compounds. In addition, an accurate mass determination is crucial in non-target screening for identifying unknown compounds. Calculating isotope peaks, which represent the natural abundance distribution of isotopes in a molecule, aids in an accurate mass determination by providing a characteristic isotopic pattern that can be used for elemental composition determination and compound identification.

Furthermore, some methods include gas chromatography (GC) or ion mobility spectrometry (IMS) as an alternative or additional separation/analysis techniques, modifying or extending the feature domains. Additional analytical dimensions significantly increase the number of data points and improve the quality of the results through additional information, e.g., clearer/ more precise substance identification. The outcoming multi-dimensional data provide characteristic fingerprints for each sample. They can be used for similarity analysis, e.g., to compare different waters or monitor individual substances’ temporal or spatial behavior based on their feature compositions [3, 8].

The NTS analytical workflow mostly aims to avoid losing any sample component at any time, resulting in very complex, multi-dimensional, and large datasets. Data processing aims to extract and convert relevant signals into substance-assigned features. However, when considering organic trace compounds, NTS data processing is challenged by reliably detecting relevant but less intense features in large, complex, and multi-dimensional datasets containing non-linear and highly variable noisy background signals. Data analysis, as the second part of NTS data evaluation, aims to compare data sets from (1) replicate measurements for filtering repeatable features, (2) different samples and blanks for prioritizing characteristic features, and (3) identify features as chemical substances.

NTA measurement data is recorded in measurement system manufacturers’ specific file formats, e.g., *.raw (Thermo Scientific), *.D (Agilent Technologies), or *.wiff (Sciex). However, exchanging or evaluating data with third-party software typically requires file format transformation to an open standard. A common format that is used in this context is *.mzXML or the newer version called *.mzML by the HUPO-PSI committee, both being XML-like files where measurement data is stored *Base64* encoded. Moreover, the data can also be compressed in several ways, e.g., *zlib* or *numpress* to significantly reduce the store size. From our observations, *zlib* reduced data size to 55 %, and adding *numpress* further reduces the size to 25 %. However, not every NTA evaluation tool can handle certain compressions; therefore, these parameter settings should be checked in advance.

In total, both parts, data processing and -analysis, are critical steps in non-target screening. They cause many challenges in NTS and hold many pitfalls due to numerous required user-defined input parameters with rarely known interactions. Therefore, this critical review presents, discusses, and makes recommendations on current NTS data evaluation to sensitize for this essential topic.

**Fig. 1:**
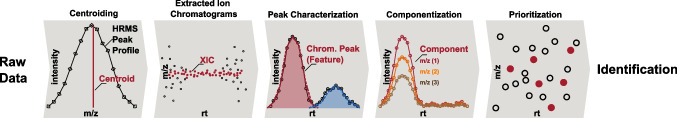
Schematic overview of a typical non-target screening data processing workflow from centroiding to prioritization

## Data processing

### Centroiding

The first user-applied data processing step in HRMS-based non-target screening is mostly centroiding the highly resolved mass profiles, which can be obtained from Fig. 1. This step significantly reduces the number of data points by a factor of 10–150, depending on the measurement system. However, different mass analyzers, e.g., Orbitrap, time of flight (TOF), or Fourier transform ion cyclotron resonance (FT-ICR), go along with different highly resolved m/z peak profile shapes, e.g., *Gaussian* (mostly for Orbitrap) [9], *Voigt*, or any asymmetric modification (may appear in TOF data) [10]. Therefore, centroiding algorithms should consider these circumstances, and in this context, it is not surprising using different algorithms may offer different results [11, 12]. To that end, using reference standards for characterizing the centroids’ mass errors is highly recommended to avoid any significant bias in this early stage of the NTS workflow. Employing reference standards ensures that the centroiding algorithms correctly account for the specific peak profile shapes and instrument characteristics, leading to a more accurate representation of the underlying data. Furthermore, the use of reference standards can enable the assessment and comparison of different centroiding algorithms, ultimately helping researchers to choose the most appropriate method for their specific analytical requirements.

#### Algorithms

In principle, centroiding extracts two pieces of information (a) position, i.e., m/z value (qualitative information), and (b) height or area, i.e., intensity value (quantitative information). Next to validated vendor-specific but often not fully accessible centroiding algorithms, the two approaches from (1) Du et al., based on continuous wavelet transform (cwt) [13], and (2) Vergeynst et al., based on the full width of half maximum (fwhm) [12], are widely spread applied and implemented in many common NTS evaluation tools, e.g., *MzMine* or *msConvert*. Both methods provide the same intensity values directly extracted from the measured data, i.e., no inter- or extrapolation. However, m/z values differ because the fwhm method, called the exact mass method, is based on interpolation, i.e., interpolation of the center within the mass peak profile’s fwhm range. In contrast, the cwt method determines m/z by local maximum analysis of the scalogram provided by cwt and, therefore, considers a measured instead of an interpolated m/z value.

Boulet et al. demonstrated that m/z errors for centroids extracted directly from measured data, e.g., using the local maximum or cwt method, can be significantly improved by interpolation approaches [11]. They presented a method involving *Savitzky-Golay’s* first-order derivative for peak detection in HRMS data in this context [14]. In principle, the algorithm detects zero crossings of the differentiated ms spectra by interpolation. This peak detection approach is not new per see; e.g., a study from John Morrey used derivate data for peak detection a long time ago [15]. However, Boulet et al. demonstrated the benefit of considering m/z errors of this method for HRMS data for the first time [11].

Next to the centroiding’s benefit of data compression, this step also goes along with information loss, e.g., peak width, which holds details about individual mass accuracy and precision [16]. Therefore, Samanipour et al. presented a centroiding method based on non-linear regression using a *Gaussian* peak model [16]. The approach called *Cent2Prof* extracts centroid information, i.e., m/z, intensity, and peak width, as regression coefficients. Moreover, they implemented machine learning (random forest) to predict peak widths considering the m/z in case HRMS profiles are not accessible. However, non-linear regression is an iterative process including much calculation effort, significantly affecting the NTS data evaluation time.

Reuschenbach et al. presented a similar approach based on regression analyses [17]. However, they followed the Caruana et al. method to reduce computation times significantly [18]. Thereby, they linearized the Gaussian function by log-transform to switch from non-linear to much faster linear regression (time save: factor 100–1000), i.e., 90’000 centroids per second. In addition to the centroids’ characteristics: of height, area, position, and width, they provided the corresponding coefficient uncertainties and a score, called Data Quality Score (DQS), that summarizes the individual centroids’ total uncertainties. The authors demonstrated that the DQS is a suitable indicator for detecting false positives in later feature lists and could improve the overall NTS workflow.

**Fig. 2:**
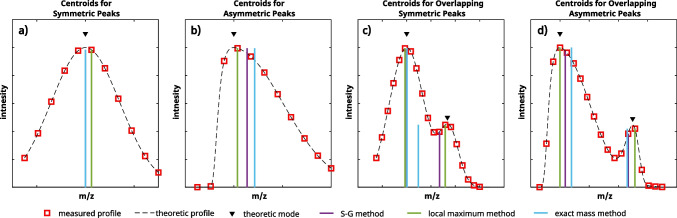
Comparison of different Centroiding algorithms for symmetric, asymmetric, isolated, and overlapping peaks: The given data is simulated to highlight the effect of m/z shifts due to asymmetry. While the main peaks for symmetric systems (a & c) provide mostly comparable centroids for different algorithms, asymmetric (b & d) as well as largely overlapped peaks, i.e., the side peak in c) may provide significantly different centroids using different algorithms

#### Challenges and Opportunities

When taking a comprehensive look at centroiding in NTS, there are three main challenges to overcome. (1) Most algorithms require user-input parameters that, not wondering, strongly influence the results. However, two effects can be distinguished: some parameters work just as filters and do not influence the centroids’ values. One example is the intensity threshold (noise level) within the exact mass method [12]. Adjusting the intensity threshold only affects the number of centroids. On the other hand, next to filters, some parameters do influence the centroids’ values, e.g., the window size of the *Savitzky-Golay* method presented by Boulet et al. [11]. Therefore, adjusting impacts the mass dispersion in a consecutive measurement of an LC-HRMS experiment and should be done cautiously. Establishing standardized criteria catalogs according to which individual parameters are selected and optimized and/or using parameter optimization routines can counteract this challenge.

(2) With some exceptions, there is less known about the reliabilities of the estimated centroids. However, such a metric could improve avoiding false positives and, therefore, increase the robustness of further evaluation and the overall cross-data comparability. Reuschenbach et al. suggested using error propagation for this purpose, a well-established approach that can be easily implemented in most existing centroiding algorithms [17].

And (3), most algorithms are affected by asymmetry properties of HRMS peaks that can occur due to physical processes, e.g., in TOF instruments, or signal overlapping, e.g., non-resolved isotopic fine-structures [10, 19, 20]. The effect of asymmetry typically leads to centroid shifts along the peak’s tailing side, which can be obtained from Fig. 2. For cwt, SG, or other convolution methods, this shifting originated from the convolution design having a moving window, and the width of this window directly influences the outcoming effect [13]. For the exact mass method (determining fwhm), asymmetry may also lead to two significantly different half widths at half maximum (hwhm) while the peak’s apex is not in the center of the full width at half maximum any longer [21]. However, without any information on the asymmetry, the exact mass method leads to m/z shifts as well. One can consider the different distances from the local maximum to the two half widths at half maximum to overcome this problem. Algorithms that consider asymmetries are clearly at an advantage since they are less susceptible to positional shifts of the centroids.

### Extracted ion chromatograms

Most NTS datasets consist of consecutive recorded HRMS spectra centroided in a former step. I.e., the number and positions of centroids are highly variable, not equidistant, and follow a continuous scale. Therefore, grouping centroids of similar m/z in subsequent spectra to obtain extracted ion chromatograms is a complex and challenging task, and several algorithms are available in this context.

#### Algorithms

Two common strategies available in many NTS evaluation tools are (1) Region of Interest (ROI) by Tautenhahn et al., which is based on the densification of mass dispersion along a chromatographic peak [22]. The ROI, i.e., XIC, is a continuous series of centroids with a low mass dispersion estimated by the empirical standard deviation. Tautenhahn et al. stated that the m/z deviation is determined by the mass accuracy of the mass spectrometer and typically increases with lower signal intensities. However, it is up to the user to define a suitable threshold. Feng et al. presented a model-based mass dispersion estimation method that is applicable to most common HRMS systems (TOF, Orbitrap, and FT-ICR) [23]. In conclusion, they proposed a more dynamic instead of a fixed thresholding approach that significantly improves the quality of the XIC obtained. The authors determined the quality by the Hoekman et al. strategy based on t-test statistics of four-fold changes using internal standard spikes [24].

(2) The Automated Data Analysis Pipeline (ADAP) provided an XIC builder alternative to ROI and was presented by Myers et al. in an NTS context [25]. ADAP sorts the whole data set by the intensity and assigns centroids to different XICs based on their m/z differences. In contrast to ROI, ADAP does not consider the retention time, i.e., strict consecutive data series are not required, and XICs may contain rt gaps. In addition and parallel to ROI, ADAP requires user input to define a mass dispersion threshold. In theory, there is no restriction to using a fixed threshold; therefore, the mentioned dynamic strategy from Feng et al. could be implemented with a manageable effort.

Zhu et al. modified the ADAP method by using Hierarchical Density-based Spatial Clustering of Applications with Noise (HDBSCAN) for assigning centroids to XICs and called the method HDBSCAN-based Pure Ion Chromatograms (HPIC) [26]. For clustering, the user must define a range for m/z and rt that limits the centroid with the highest intensity, called anchor ion. Then, the HDBSCAN is applied within this ranged two-dimensional space, and the clustered centroids are deleted from the original data set. Finally, the procedure is repeated with the remaining centroids until an intensity threshold is subceeded. The HPIC is not limited to constant m/z traces but also allows first- or second-order linear trends in those traces. Moreover, the user-defined ranges should not affect the XICs and are mainly used to reduce calculation efforts. The HDBSCAN does not require user input, so HPIC can be seen as a parameter-free XIC builder.

#### Challenges and Opportunities

In parallel to centroiding, the XIC building step reveals similar challenges: (1) different algorithms provide different XICs. Therefore, it seems unsurprising that Hohrenk et al., when comparing four different software tools for NTS data evaluation, only obtained a 10 % overlap of the detected features [27]. On the other hand, Zhu et al. presented a larger overlap of 60 % but compared only three different workflows [26]. Therefore, both studies are not directly comparable. The following problematic question emerges: When can XICs be assumed to come from the same population and thus contain the same feature? One approach to address this issue is to estimate the confidence intervals of the XICs, which can measure the uncertainty and variability associated with the values. By leveraging the bootstrap method, a widely used non-parametric technique for estimating confidence intervals, we can better understand the reliability and comparability of the XICs from different algorithms. This would also directly preempt the second challenge.

(2) The qualities or reliabilities of XICs are rarely reported and, even more concerning, not considered within further processing. Especially considering reliabilities would offer a smart opportunity for later result prioritization. However, a promising approach already exists for characterizing the clustering’s output: the silhouette score, which considers the distances of a centroid within its cluster and the distances to the neighboring cluster. I.e., the larger the relative gap between clusters, the more reliable the XIC assignment and the better the silhouette score [28].

In NTS, this silhouette score is used for optimizing parameters in the XIC building, which can be obtained from Wei et al. [29]. Their study presented a DBSCAN-based XIC builder similar to the later proposed HPIC from Zhu et al. [26], and the DBSCAN parameters were obtained from silhouette score-based optimization. However, the suggested further score reporting and consideration for later result prioritization are not yet implemented in NTS XIC builder algorithms. From a broader perspective, Starczewski et al. further improved the silhouette score by adding a normalization term [30]. The new criterion, called SILA, allows for comparing clusters more robustly and would open the opportunity to measure the quality of individual XICs, which is of interest for further prioritization or optimizing the analytical measurement method.

### Chromatographic peak characterization

In many applications, XIC building and characterization of the chromatographic peaks are directly combined. However, even though these are two successive steps, both have different algorithms, challenges, and opportunities. On a basic level, the numerical task for peak characterization is very similar to centroiding HRMS peaks. However, the peak area is more relevant than the signal intensity for centroiding due to the proportional relationship between peak area and concentration that persists even with asymmetric peaks. Therefore, it is more important to accurately and robustly detect the chromatographic peak edges or integration limits. Moreover, within this step, the m/z value for each peak is estimated, which makes this step very critical for further feature identification/annotation.

In general, XICs can be divided into four different categories: containing (a) a single peak, (b) sufficiently resolved and therefore multiple isolated peaks, (c) insufficiently resolved and therefore overlapping peaks, and (d) the absence of any peak. While category (a) is the best for accurate and robust peak determination, it has to be mentioned that XICs of the other categories may occur due to insufficient XIC building and instrument limitations, i.e., co-elutions.

#### Algorithms

The cwt method is one of the most common algorithms for peak characterization in NTS, and Tautenhahn et al. implemented it in their centWave algorithm [22]. Local minimum ridges of the cwt coefficients within the scalogram are considered for determining the peak edges. This is a robust strategy since the cwt also acts as a noise-reducing smoothing filter by design. However, the proposed proportional relation from Du et al. between the cwt coefficient at the peak apex and the peak area is not considered by centWave [13]. Instead, ordinary trapezoid integration within the peak range is used. However, this modification avoids asymmetry effects on the cwt coefficients, which would lead to a bias in this context. In addition, the m/z value is estimated using an intensity-based weighted average of the m/z centroids within the peak range.

Curve fitting using Gaussian or asymmetric modifications is another technique for chromatographic peak detection. In this context, performing a non-linear regression using a Gaussian function is the most intuitive. This option is offered by the *centWave* algorithm as an alternative to ordinary trapezoid integration [22]. Furthermore, if multiple peaks occur in an XIC, the sum of multiple Gaussian functions is used. I.e., curve fitting with multiple components is a deconvolution process; therefore, it is possible to characterize overlapping peaks more accurately.

However, Gaussian functions are symmetric, while this restriction is not true for chromatographic peaks, and to that end, the exponentially modified Gaussian function (EMG) is often used in chromatography. Optimization of the separation method is limited for NTS due to the unknown and highly variable sample composition [31]. Isaacman-VanWertz et al. presented an automated non-linear regression method for LC-HRMS that considers both Gaussian and exponentially modified Gaussian for peak characterization [32]. The authors obtained comparable results to other methods. However, as an additional benefit, this method offers statistical parameters, e.g., the uncertainty of the individual peak areas or retention times.

Furthermore, the proposed algorithm can also handle XICs that contain only noise. The authors implemented a looping approach in which the dataset was repeatedly smoothed and subjected to peak detection. Each iteration increased the smoothing span if no peaks were identified. Once peaks were detected, their characteristics, such as regression results and statistical metrics, were analyzed to effectively separate relevant peak signals from background noise. However, the disadvantages of non-linear regression are (1) the required initial fit coefficients that can significantly influence the results and (2) the calculation effort, as non-linear regression is an iterative process. Moreover, there is no separate calculation of the m/z, and this value needs to be estimated in advance.

Wei et al. presented a similar strategy using different regression models for peak characterization [29]. They included six different non-linear peak functions and used a retention time-dependent training approach based on the most intense isolated peaks to decide which model fits best in discrete rt ranges. Therefore, calculation efficiencies should be slightly improved compared to Isaacman-VanWertz et al. because regression is performed only once for most peaks.

Brotons et al. also presented a regression-based peak characterization called Pipelines and Systems for Threshold-Avoiding Quantification (PASTAQ) [20]. PASTAQ combines centroiding, extracted ion chromatogram building, and chromatographic peak characterization. Therefore, the algorithm works as a two-dimensional peak regression. Brotons et al. used, like Reuschenbach et al., the Caruana approach for linearizing the Gaussian function, but in PASTAQ, it is performed for the rt and m/z domains simultaneously. PASTAQ is still very fast, even considering many data points due to linear regression. However, the Caruana method cannot handle overlapping or asymmetric peaks because it is no deconvolution method for mathematical reasons (logarithm laws), and no linearized asymmetric peak function is known yet. A bias based on overlapping peaks cannot be avoided with this approach. The m/z value is indirectly extracted from the regression coefficients and weighted by the signal intensity. The latter is due to the linearized regression using logarithms performed as a weighted regression.

**Fig. 3:**
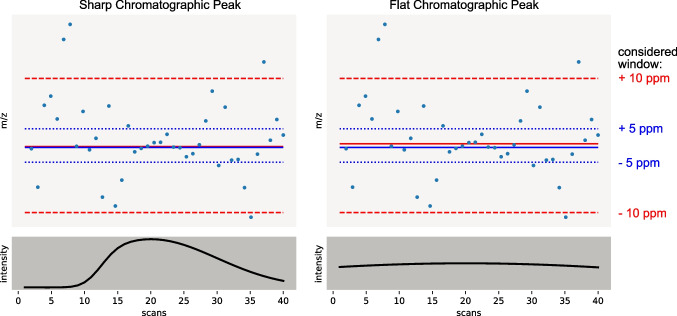
Effect of peak shape and m/z dispersion window on the calculated intensity-weighted mean for m/z of a chromatographic peak. The example data given is based on a simulation to highlight the effect. For both cases, sharp and flat, the mass dispersion window slightly changes the m/z calculated, which can be obtained from the horizontal straight lines (blue: 5 ppm; red: 10 ppm). In this context, the solid lines (**—**, **—**) provide the resulting m/z values, while the dotted lines ($\cdots$) provide the borders of the 5 ppm selection window and the dashed lines (- - -) provide the respecting borders of the 10 ppm window. The peak profile also influences the effect strength, which can be obtained by comparing the spread of the two lines for the sharp peak (left) and the flat peak (right)

Dietrich et al. presented an empirical peak characterization method that adapts a manual peak characterization, but they realized this in an automatic workflow [33]. The algorithm detects all local maxima and the corresponding peak boundaries via an iterative loop along the intensity profile until a defined threshold is subceeded. Furthermore, Peaks are split by valley points or merged if the apex-valley ratio falls below a defined threshold. For the m/z, Dietrich et al. considered the intensity-weighted average of the corresponding m/z values between the peak boundaries. Besides the user-input parameters, this method is one of the most intuitive and time efficient.

#### Challenges and Opportunities

One of the main challenges in chromatographic peak characterization is accurately and robustly detecting the number of peaks within an XIC. On the one hand, all XICs containing only noise are false positive candidates. However, on the other hand, all peaks not detected are false-negative results. This contradiction ends up in a dilemma; decreasing false negatives also means increasing false positives [34]. To that end, a common strategy is paying the price of additional false positives and using filters to delete these afterward. Adding a user-defined threshold parameter is then a commonly used approach.

However, as there is no suitable feedback or target value to characterize the quality of a discrete parameter setting, the optimization strategy needs to be improved. In this context, Dietrich et al. avoid optimization by estimating the signal-to-noise ratio for every peak, a normalized parameter with well-established thresholds, e.g., s/n > 3 or s/n > 10 [33]. With this, the noise amplitude is often estimated directly next to the corresponding peaks, i.e., beyond the peak boundaries. However, since noise cannot be extracted from all XICs in that way, this method is unsuitable as a general strategy. In particular, for Orbitrap-MS data, XICs with single chromatographic peaks rarely contain data points beyond their peak boundaries.

Next to s/n, the peak width, including minimum and maximum limits, is another commonly used filter criterion [22, 33]. A lower limit can be justified by the minimum number of points required to describe a Gaussian peak. From a statistical perspective, this limit is four due to the three parameters height, width, and position, which result in one degree of freedom. The higher limit can also be deduced by applying a model; in this context, peak broadening in chromatography is a well-known field [35]. However, in many algorithms, this information is not considered; instead, the user should define fixed values for upper and lower limits, which may decrease the comparability between different studies.

Using regression for peak characterization, e.g., PASTAQ [20] or the non-linear regression by Isaacman-VanWertz et al. offers a promising filter approach based on the regression confidence [32]. The better the quality of the original data, the lower the uncertainty of the output coefficients. The adjusted coefficient of determination (${\bar{R}}^{2}$) is a standard statistical parameter. However, since there is no generally accepted minimum value for ${\bar{R}}^{2}$, this decision is left to the user. For the linearized regression approaches, a global F-test would be one promising candidate to resolve user dependency. Thus, peak models are only considered if they significantly improve over a baseline signal with a constant offset. Therefore, this decision is based on generally accepted p-Value limits of 0.05.

Melnikov et al. presented a machine learning approach based on convolutional neural networks (CNN) to classify XICs [36]. Their algorithm, called peakonly, defined three classes: (1) no peaks, (2) one or more peaks, and (3) complex peak-like signal that requires manual expert inspection. As a result, they obtained that 32 % of the features detected by XCMS online were classified as noise and 17 % as complex/uncertain peaks. A significant benefit of employing machine learning algorithms is their reduced reliance on user intervention or input. However, those approaches highly depend on the training data, i.e., applying machine learning by another research group having different training data leads to different results. Moreover, machine learning is still a red flag for many users and requires extensive background knowledge to assess and interpret the results obtained.

Another challenge in NTS peak characterization is determining the m/z value. In this context, intensity weighting is often used to avoid influences of data points at the peak edges with larger mass dispersion but are less intense. However, for flat peaks, i.e., low height/width ratio, this weighting may not compensate for m/z distortion, leading to an increased mass deviation in replicate measurements, which can be obtained from Fig. 3. On the other hand, high signal intensities are no guarantee for accurate and precise m/z values, as this assumption is based on empirical findings. Depending on the measurement instrument, extrema like high intensity-high mass dispersion and low intensity-low mass dispersion can also be obtained in the NTS dataset. To that end, reporting the uncertainties of the estimated m/z values is urgently needed. Statistical concepts for the uncertainty of weighted averages are well-established but, unfortunately, not yet implemented in many weighted average m/z estimations.

### Alignment and componentization

In modern NTS data evaluation, multiple features originating from the same chemical substance, e.g., isotopes, adducts, fragments, or cluster ions, are grouped as a component [37]. Grouping these features compresses and structures the data set and later be used for substance identification [38]. However, it has to be mentioned that the term component is not harmonized, and other notions, like molecular feature, bucket, feature, or compound, exist in parallel. In addition, componentization also partially involves grouping features detected across multiple samples, e.g., by replicate measurements or blank correction. However, data sets, especially rt, vary from measurement to measurement for a certain amount across-sample calculations, i.e., data comparisons require an alignment step for rt and m/z in advance.

#### Algorithms

Kuhl et al. published the CAMERA package, one of the most common approaches for componentization in NTS [39]. In short, the algorithm considers multiple chromatographic peaks as a component if they have (1) similar elution profiles within one sample [40], (2) correlating intensities across samples [41], and (3) chemically plausible m/z differences. All criteria are used to calculate a score that is further considered in cluster analysis for grouping. Isotopes and common adducts are annotated by comparing the intensity ratios or m/z differences with a reference database. The CAMERA algorithm does not require user input parameters directly, as all information is extracted from the data set and feature list. However, as the algorithm considers across-sample correlations, an alignment process is required to harmonize rt and m/z along multiple measurements.

Permiakova et al. presented a componentization strategy fully based on XIC similarity called Chromatogram HIerarchical Compressive K-means with Nyström approximation (CHICKN) [42]. First, the authors used the Wasserstein distance to characterize the pairwise similarity between two XICs by their cumulative intensity profiles. Then, they transformed the similarities with a Kernel-based mapping from an rt-m/z into a feature space where similar features are close. Finally, a K-means cluster analysis is used for componentization. As an advantage, this algorithm works with XICs within and across samples and is, therefore, more flexible than CAMERA. However, chemical criteria, e.g., isotope ratios or common known m/z differences, are not considered.

Whenever across-sample consideration is made, alignment of the XICs or features is required due to natural variations. One very intuitive way to do this is based on simple tolerances for the individual domains, mainly m/z and rt [43]. However, these approaches assume variations to be constant or linear scaled, strongly depend on the user input, and often do not provide feedback on whether the settings chosen are suitable.

Prince and Marcotte developed an alignment algorithm based on dynamic time warping called ordered bijective interpolated warping (OBI-Warp) [44]. The algorithm analyses the similarity of mass spectra across samples by their correlation and creates a similarity matrix. Then, the path with the best similarity scores from the lower left (first spectra) to the upper right (last spectra) is extracted for warping. In this context, the deviation from the path extracted to the diagonal of the similarity matrix is considered for rt correction. In contrast to the abovementioned approach, OBI-Warp conserves the mass spectra, which is a chemically meaningful strategy, ensuring the same measurement conditions for centroids recorded at once. However, this alignment requires a much higher calculation effort due to similarity matrix calculation and the nature of a pairwise approach. Moreover, OBI-Warp also includes some user parameters to be optimized.

Gorrochategui et al. developed a componentization strategy based on the region of interest and multivariate curve regression (ROIMCR) [45]. The presented algorithm does not require a peak characterization step and provides calculated mass spectra of m/z traces with similar elution profiles. Therefore, ROIMCR differs from most NTS evaluation workflows that typically provide feature lists based on individual XIC peak characterizations. Furthermore, by providing mass spectra, which can be later used for compound identification, there is no need for any alignment. Moreover, the ROIMCR algorithm is developed for single-sample and multi-sample componentization. The main principle of an MCR is a matrix decomposition by applying a bi-linear regression. In this context, the XICs are provided by the ROI approach and are transferred into a matrix. This matrix containing intensities at specific rt (row) and m/z (column) is decomposed into submatrices that contain information on calculated intensity profiles along rt (submatrix 1) and calculated mass spectra (submatrix 2). The critical parameter is the number of components explaining most of the data set’s variance. If this value is chosen too small, it has some effect on the overall result. On the other hand, a value that is too large can lead to overfitting, which can also affect the results. Therefore, an iterative optimization approach is commonly used, but as matrix decomposition on large datasets demands a high calculation effort, ROIMCR is time-consuming.

#### Challenges and Opportunities

The alignment across samples is critical; unfortunately, this step is mainly based on user-input parameters, and therefore, there is always a risk of decreasing the comparability between studies [27]. In this context, the first steps have already been taken to improve comparability. For example, Aalizadeh et al. propose a harmonized rt index for the NTS [46].

A relevant challenge for alignment and componentization is connected to the similarity analysis of XICs. Most approaches in NTS used for this task require XICs of the same length, e.g., to calculate the correlation of two or more elution profiles. Those considerations are based on a point-by-point concept and only work if every point from one profile can be assigned to a certain point in the other. However, NTS signals detected are not arranged in a predefined discrete grid but are stored data-dependent as individual coordinates in a continuous rt, m/z, and intensity space. XIC building does not remove this continuum space. Still, a discrete space is required for the alignment algorithms used in NTS, which is solved by a gap-filling process that adds a bias to the results.

Genolini et al. developed a more general concept that was not optimized for NTS but also covered the numerical alignment task. Thereby, the authors consider grouping curvatures using longitudinal clustering [47]. In this approach, called kmlShape, the Fréchet distance describes the similarity of different curves, i.e., XICs. The main advantage of using Fréchet distance is that the number and positions of points within these curves can differ. On a basic level, kmlShape is a K-means clustering using the Fréchet metric that considers curvatures’ shape. In the context of NTS data, kmlShape could group XICs across and within samples by their elution profile similarity. Moreover, the method would provide average elution profiles for m/z traces across samples.

### Prioritization

In most cases, an NTS data processing workflow provides some feature list that contains at least rt, m/z, and intensity information for single or multiple samples. However, a prioritization for further analysis is highly recommended due to the sheer number of 1000–10,000 features. Therefore, this step extracts the most characteristic, relevant, or concerning features.

#### Algorithms

A common prioritization strategy is considering the intensity-based fold change of a feature across samples, e.g., as presented in a study by Schollée et al. [48]. The fold change is defined as the ratio between two values typically transformed into a log-2 scale, i.e., a fold change of 1 means the intensity is doubled, while -1 means the intensity is halved. User-defined thresholds are then used to extract features with significant absolute fold changes. However, the fold change is often combined with a significance descriptor, e.g., a p-value obtained from t-tests, ANOVA, or other statistical tests [8]. As an advantage, the p-value has an established threshold of 0.05.

Köppe et al. presented a practical approach to removing ubiquitously found features when analyzing wastewater [49]. The general principle is defining and excluding the intersecting features across samples. Therefore, the authors identified features that occur in most samples by defining a threshold (80 %). Using this strategy, they significantly reduced the number of features by an additional 30 %. The method helps identify features that originate from a specific source. However, relationships between features like (anti-) correlations are not considered.

Purschke et al. developed a multivariate method to prioritize features by applying principal component analysis (PCA) to their data set that consists of a time series NTS monitoring [3]. In the PCA, they analyzed the variation of the time series (variables) for all features (objects). According to this, the authors obtained scores describing each feature’s position in the multivariate space, where similar scores reflect similar features. Besides, the PCA provides the loadings that describe the different time series trends. Based on the scores and loadings, they could distinguish relevant features with increasing or decreasing trends from less-relevant fluctuating features. However, in parallel with alignment and componentization, such matrix-based strategies require data having no gaps to perform best. If this is not the case, e.g., some feature is not detected or miss-evaluated at some point within the time series, the gap filling adds some bias to the results.

#### Challenges and Opportunities

The prioritization step significantly reduces the features and should be performed carefully because miss-prioritization may add blindspots to the analysis. Most of the algorithms used for this task are based on the features’ variance. However, the problem with variances is that precise, representative, and repeatable results require a large sample set. The more samples are included, the more unique features are added to the lists. In contrast, feature space is limited due to the m/z and rt ranges of the measurement method, which implies that overall similarities decrease. Therefore, prioritization is always a compromise, and it strongly depends on the sample set, which reduces the result comparability between studies by design. Overall, prioritization is certainly one of the most far-reaching steps in NTS data processing, and the concepts presented here show only a small section.

**Table 1:** Overview of the algorithms discussed in this paper: merged cells indicate that the corresponding algorithm covers multiple aspects of NTS data processing

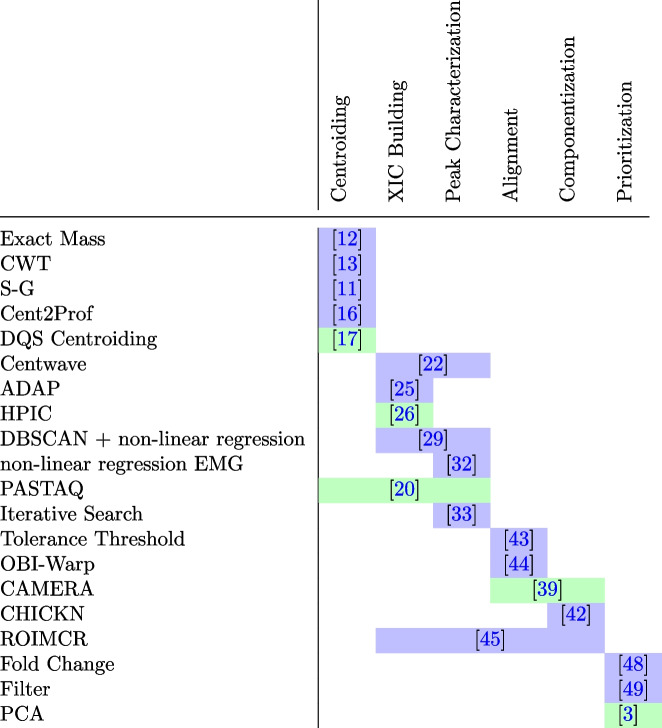	

The background color indicates if the algorithm requires user-input parameters (green: no user parameters must be defined)

## Conclusion

The methods presented in this article underline that NTS data evaluation is (1) a complex multi-step process with a continuously increasing toolbox of algorithms [50], each with its unique strengths and weaknesses, and a non-comprehensive but common selection can be obtained from Table 1. (2) NTS is still very challenging, especially when considering the comparability and reliability of results [25, 27]. Looking at the data processing workflow in total, it is striking that the current challenges have some commonalities. For example, many algorithms are heavily dependent on user input parameters. However, in this context, a promising approach to this problem results from automated parameter optimization [51], such as the isotopologue parameter optimization IPO by Libiseller et al. or Autotuner by McLean and Kujawinski [52, 53].

Some studies show that many user input parameters are unnecessary and work with standardized statistics instead. Centroiding by Reuschenbach et al., the XIC building by Zhu et al., and Prioritization by Purschke et al. are good examples in this context (Table 1) [3, 17, 26]. All these algorithms avoid human-based subjectivity in the NTS data evaluation and, therefore, may increase the cross-study comparability. However, this promising hypothesis was not yet tested.

Unfortunately, there is much potential uncovered yet, because the uncertainty connected to all intermediate results is partially obtained, less reported but not taken into account by the further processing steps. Thus, the uncertainty of a centroid in m/z does not play a role in assembling an ion chromatogram, although increased uncertainties may indicate a false positive feature [17]. So, at this point, future workflows should collect the uncertainties of intermediate results throughout the process and incorporate them into the prioritization of features at the end, e.g., to improve the further result interpretation step. Especially under the aspect of comparability of results, a good estimation of the confidence intervals of individual features is very helpful.

Filtering false positive features itself is still a major challenge, as it requires information that is often not available: (1) is the data quality sufficient to make a reliable statement; if not, there is probably a false positive feature, and (2) can the feature be traced back to a plausible chemical compound. For the first point, the Schymanski score checks at least the availability of reference data to reflect the reliability of a feature [38]. However, the raw data quality is hardly considered. Here as well, workflows should be improved with regard to the information on confidence intervals that include the entire data evaluation process. The second point is primarily up to the NTS community in total. Open-access data exchange services, e.g., the Digital Sample Freezing Platform (DSFP), should be used more frequently [54].

To advance the field of NTS data analysis, the following future perspectives are proposed for developers of NTS data processing algorithms and workflows:

1. Minimize user input parameters: Focusing on developing algorithms that require minimal user input parameters can reduce human-based subjectivity in NTS data evaluation. This potentially increases the cross-study comparability and reduces biases introduced by arbitrary parameter selection. An automated parameter optimization is a promising approach in this direction, as demonstrated by existing studies [51–53].
2. Address uncertainties and data quality: Incorporating the uncertainties associated with intermediate results and raw data quality into the data processing algorithms is essential for filtering false positive features and ensuring reliable outcomes. Developers should work on creating methods that consider these uncertainties and offer reliable ways to evaluate the data quality at every step of the processing workflow [17, 38].

Moreover, the following future perspectives are proposed for users of NTS data processing algorithms and workflows:

1. Characterize the strengths and weaknesses of chosen algorithms/workflows: It is essential for users to understand the strengths and weaknesses of the algorithms and workflows they choose. A comprehensive and detailed report on NTS data evaluation should be prepared to ensure traceability and provide a better understanding of the results obtained, as well as their reliability and limitations [3, 17, 26, 51–53].
2. Utilize open-access data exchange platforms and community resources: To enhance the accuracy and traceability of detected features, users should leverage open-access databases, data exchange platforms, and community resources. Sharing data and collaborating with the NTS community enables validation, better interpretation of results, and a more accurate understanding of the underlying chemistry [38, 54].
